# Alexithymia and Psychopathology in Patients Suffering From Inflammatory Bowel Disease: Arising Differences and Correlations to Tailoring Therapeutic Strategies

**DOI:** 10.3389/fpsyt.2018.00324

**Published:** 2018-08-03

**Authors:** Caterina A. Viganò, Marta M. Beltrami, Monica F. Bosi, Riccardo Zanello, Marta Valtorta, Giovanni Maconi

**Affiliations:** ^1^Biomedical and Clinical Sciences Dept. L. Sacco, Università degli Studi di Milano, Milan, Italy; ^2^Psychiatry Unit, Biomedical and Clinical Sciences L. Sacco, Università degli Studi di Milano, L. Sacco Hospital, Milan, Italy; ^3^Psychiatry Unit 2, ASST Fatebenefratelli Sacco, Milan, Italy

**Keywords:** psychopathological symptoms, ulcerative colitis (uc), alexithymia, integrated care, crohn disease

## Abstract

Comorbidity with anxiety or depression is common in patients with Inflammatory Bowel Disease (IBD) as Crohn Disease (CD) and Ulcerative Colitis (UC). Data suggest that the cognitive construct of alexithymia has high prevalence in people suffering from anxiety and mood disorders and even in people with IBD. Most studies have investigated mainly anxiety and depression, considering IBD population as a homogeneous group of patients. Little evidence shows the impact of alexithymia on the course of IBD. We evaluated a broad spectrum of psychopathological symptoms and alexithymia levels in a group of outpatients affected by IBD in clinical remission, comparing CD and UC and investigating the relationship with clinical and socio-demographic variables. One hundred and seventy IBD outpatients were screened by using the Hospital Anxiety Depression Scale (HADS), the Self-report Symptom Inventory-90-Revised (SCL-90-R) and the Toronto Alexithymia Scale (TAS-20). A high prevalence of anxious and depressive symptoms (42.35 and 25.8% respectively) together with alexithymia (31.76%) was confirmed. CD patients experienced high levels of depression (HADS Depression 35.2% *p* = 0.034; SCL-90-R mean 1.39 *p* < 0.001), somatisation (SCL-90-R mean 1.04 *p* < 0.001), obsessive-compulsive symptoms (SCL-90-R mean 1.2 *p* < 0.001), and global severity (SCL-90-R mean 1.15 *p* < 0.001). There is no statistical difference in the prevalence of alexithymia in both subpopulations. The levels of alexithymia are correlated to the levels of anxiety (HADS Anxiety rs = 0.516 *p* < 0.001), depression (HADS Depression rs = 0.556 *p* < 0.001; SCL-90-R rs = 0.274 *p* = 0.001), somatisation (SCL-90-R rs = 0.229 *p* = 0.005), obsessive-compulsive symptoms (SCL-90-R rs = 0.362 *p* < 0.001), and global severity (SCL-90-R rs = 0.265 *p* = 0.001). Furthermore, alexithymia is associated with a delay of diagnosis of IBD, poly-therapies and greater IBD extension. Older age, female gender, greater IBD extension, surgery, and delay of diagnosis seem to be related to a high prevalence of psychopathological symptoms such as anxiety, depression, somatisation, and obsessive-compulsive symptoms. Psychopathological symptoms and high levels of alexithymia are frequent in IBD patients and seem to be related to a high risk of poor clinical outcome. CD patients could be considered at higher risk of mental comorbidity. A more comprehensive psychiatric assessment, including alexithymia, and an integrated treatment of underlying conditions, must be taken into account in order to improve the global prognosis of the disease.

## Introduction

Chronic Inflammatory Bowel Disease (IBD) mainly represented by Crohn Disease (CD) and Ulcerative Colitis (UC), are characterized by the occurrence of chronic inflammation with an unpredictable evolution distinguished by flares and remissions. While inflammation in CD can involve the entire gastrointestinal tract (1), the inflammation in UC is localized at the colon and rectum level only. The incidence of IBD in Italy is comparable with that reported in Northern Europe and has gradually increased in the last decades (9.6/100.000 for UC and 3.4/10.000 for CD) (2). Patients who suffer from IBD frequently manifest extra-intestinal complications and need to undergo surgical conservative therapy or resective surgery, with different procedures between CD and UC (3, 4). The IBD pathogenesis is multifactorial: environmental factors can give rise to an inappropriate and excessive intestinal immune response in genetically-predisposed subjects and in the presence of dysbiosis of the gut microbiota (5). The chronic nature of IBD and its frequent onset in early adulthood (15–30 years old) (6) cause severe stress and worsening of quality of life (4, 7). Multiple flares, low social support and high stress levels, as well as psychiatric comorbidities, usually worsen the quality of life (8) and increase the risk of mental distress when compared to the general population (9, 10). A substantial quantity of data showed the comorbidity among anxiety, depression and IBD, whose prevalence is estimated to be around 30% in patients in clinical remission, while it reaches up to 60–80% during the active phase of the disease (11–13).

Several studies identified the risk factors causing anxiety and depression in IBD. The female gender (14), old age, high levels of perceived stressed influenced by negative coping strategies (15, 16), active or severe diseases (17), surgical complications and low-level of perceived social support (18, 19) seem to be the main risk factors for the development of psychiatric symptoms in IBD patients (20–22). The presence of anxious or depressive symptoms is associated with reduced treatment compliance (23), increase in disability (24), and mortality (25).

Epidemiological studies highlighted a high prevalence of specific psychological functions such as alexithymia among patients suffering from anxiety disorders (26), obsessive-compulsive disorders (27), and mood disorders (28, 29), even in patients affected by chronic and immune-mediated pathologies with somatic symptoms (30).

Alexithymia is a construct characterized by specific cognitive traits like the difficulty in identifying, describing and differentiating emotions, with a reduction of imaginative processes (poverty of imagination and fantasy) and with a cognitive style oriented to the external reality and to operative thinking (31). The prevalence of alexithymia in patients suffering from depressive disorders reaches 33.3%, while it is around 42% in patients suffering from anxiety disorders (32). These rates of comorbidity suggest a correlation between the phenomena; data assume that alexithymia may interfere with response to treatment in depressed and anxious patients (33). Even in IBD patients the prevalence is about 35% (34); it is well-known that alexithymia is closely related to clinical severity of gastrointestinal functional disease (35). Some studies identify alexithymia as a stable construct not influenced by the clinical evolution of gastrointestinal pathologies (36) with a negative impact on the subjective health status of IBD patients (37).

To date, most studies have mainly investigated the prevalence of anxiety and depression considering IBD as a homogeneous group of patients (38); little data considered the two sub-populations of UC and CD patients independently (12, 13) and evaluated the association between IBD and specific psychological functions (39). However, so far no study has evaluated a broader spectrum of psychopathological symptoms. This evidence suggests that psychopathological comorbidity could be under-recognized and under-diagnosed in clinical practice with standardized patterns of care, which do not consider the possible specific different needs and personal outcomes. Indeed, a recent survey identified that in the US only 36% of patients affected by IBD and depressive symptoms actually received professional support (40).

The aim of this study is to evaluate a broad spectrum of psychopathological symptoms, psychological distress, and alexithymia levels in a group of Italian outpatients affected by IBD in clinical remission, analyzing possible differences between the two subpopulations of patients (CD and UC) and investigating possible links to specific clinical and socio-demographic variables.

## Materials and methods

### Participants

In this study, a consecutive series of IBD outpatients was recruited from January 2016 to September 2017 and regularly followed-up at the IBD Centre of the Gastroenterology department and IBD Unit of the local public healthcare facility ASST-Fatebenefratelli-Sacco University Hospital in Milan, Italy. All patients met the inclusion and exclusion criteria reported in Table 1.

**Table 1:** Inclusion/exclusion criteria.

Inclusion criteria: Diagnosis of Crohn's Disease of Ulcerative Colitis in clinical remission (Crohn's Disease Activity Index (CDAI) < 150, Mayo score 0–2). Age between 18 and 65 years old Informed consent given by the patient to take part in the study.
Exclusion criteria: Crohn's Disease or Ulcerative Colitis in active phase Previous psychiatric diagnosis IQ < 70 Active pregnancy, cognitive impairment, documented head trauma, concomitant neurological, or oncologic pathologies Comorbidity with alcohol or substance abuse or severe personality disorder

### Study design

The eligible sample was screened considering the following variables:

- Socio-demographic and clinical variables through a standardized survey (diagnosis, gender, age, marital status, level of education, employment status, therapy, family history of IBD, age at onset of IBD symptoms, age at diagnosis, diagnostic delay, disease extension, surgery).
- Psychological distress screening through the Hospital Anxiety and Depression Scale (HADS) (Cronbach's alpha 0.83 for HADS-Anxiety, 0.82 for HADS-Depression) (41, 42).
- Psychiatric symptomatology assessment through the Self-report Symptom Inventory Revised (SCL-90-R) (Cronbach's alpha 0.96) (43, 44).
- Alexithymia levels evaluation through the Toronto Alexithymia Scale (TAS-20) (Cronbach's alpha 0.81) (45, 46).

### Statistical analysis

The distribution of the obtained data was evaluated, and resulted in having non-parametric characteristics. For the anxious-depressive symptomatology-screening test (HADS), for the psychiatric symptoms evaluation test (SCL-90-R), and for the test concerning the alexithymia dimension (TAS-20) the mean scores were evaluated in the total population of patients and in the sub-populations of patients affected by UC and CD. As far as the HADS and TAS-20 scales are concerned, the prevalence of resulting positive according to the decisional cut-off values for each sub-scale was also considered (HADS score ≥ 8 for the anxiety or depression scale, TAS-20 score ≥ 50, including also borderline patients) (42, 47). The values of the UC and CD groups were compared using the Wilcoxon-Mann-Whitney test, considering values of *p* < 0.01 as significant. The relation between symptomatology, alexithymia level, and socio-demographic variables were studied through the Spearman's Rho correlation (rs), considering values *p* < 0.01 as significant, while values of *p* < 0.05 were considered as significant in some cases of data considered of clinical interest. The statistical analysis was carried out by using the SPSS program, version 24.

## Results

We sequentially recruited 170 patients who met the study inclusion criteria; 68 patients suffering from CD and 102 suffering from UC. The related socio-demographic and clinical characteristics are described in Table 2.

**Table 2:** Socio-demographic and clinical variables.

Diagnosis, n (%)	Crohn's disease (CD): 68 (40%) Ulcerative colitis (UC): 102 (60%)		
Gender, n (%)	Male: 94 (55.3%) Female: 76 (44.7%)		
Age (years) mean (± SD)	47.1 (±12.03)		
Marital status, n (%)	Married: 80 (47.06%) Single: 67 (39.41%) Widowed: 15 (8.82%) Divorced: 8 (4.71%)		
Level of education, n (%)	High school: 85 (50%) Middle school: 40 (23.53%) University degree: 40 (23.53%) Elementary school: 5 (2.94%)		
Employment status, n (%)	Employed: 105 (61.76%) Self-employed: 33 (19.42%) Unemployed: 14 (8.23%) Pensioner: 13 (7.65%) Student: 5 (2.94%)		
Therapy, n (%)	NSAID: 105 (61.76%) Immunosuppressive medications: 38 (22.36%) TNF inhibitor: 14 (8.23%) Methotrexate: 5 (2.94%) Poly-therapy: 8 (4.71%)		
Family history of IBD, n (%)	Yes: 35 (20.59%) No: 135 (79.41%)		
Age at onset of IBD symptoms (years) mean (± SD)	29.72 (±10.48)		
Age at diagnosis (years) mean (± SD)	30.3 (±10.02)		
Diagnostic delay (months) mean (± SD)	11.8 (±30.54)		
Extension of IBD, n (%)	UC		CD
	Proctitis: 8 (7.8%)		L1: 4 (5.9%)
	Left side colitis: 32 (31.4%)		L2: 40 (58.8%) L3: 24 (35.3%)
	Subtotal or pancolitis: 62 (60.8%)		L4: 0
Surgery, n (%)		UC	CD
	Yes	20 (19.6%)	38 (55.9%)
	No	82 (80.4%)	30 (44.1%)

### Prevalence of anxious and depressive symptoms and general psychopathological symptoms

Considering the total population undergoing screening, around 42.35% of patients resulted having positive scores for anxious symptoms and 25.8% for depressive symptoms. The prevalence of anxiety was comparable between the two groups (42.15% in UC and 42.63% in CD) while the prevalence of depressive symptoms was greater in CD patients (35.2% vs. 20.5% *p* = 0.034).

Crohn's disease patients had greater scores of somatisation (mean 1.04; ± SD 0.92 *p* < 0.001), obsessive-compulsive symptoms (mean 1.2; ± SD 1.17 *p* < 0.001), depression (mean 1.39; ± SD 1.09 *p* < 0.001), and global severity index (mean 1.15; ± SD 0.75 *p* < 0.001), as shown in **Table 4**.

### Alexithymia levels in the sample

The prevalence of alexithymia in the total sample is 31.76%, 36.8% in CD and 28.43% in UC patients, with no statistical differences between two groups (*p* > 0.05) (Table 3).

**Table 3:** Prevalence of positive tests (HADS Anxiety ≥ 8, HADS Depression ≥ 8, TAS – 20 total score ≥ 50) in a comparative analysis between UC and CD using Wilcoxon-Mann-Whitney test (*p* < 0.05).

	**Total**	**UC**	**CD**	**U**	**W**	**Z**	***P***
HADS Anxiety % (n)	42.35% (72)	42.15% (43)	42.63% (29)	3397.5	8753.5	−0.197	0.843
HADS Depression % (n)	25.8% (45)	20.50% (21)	35.2% (24)	2958	8211	−2.123	0.034
TAS-20 total score % (n)	31.76% (54)	28.43% (29)	36.80% (25)	3222.5	8373.5	−1.031	0.303

The mean values for alexithymia in the global population were significant when taking into consideration the dimensions defined as “externally-oriented cognitive style” (mean 19.89 ± SD 7.48) and “difficulty in identifying feelings” (mean 15.12 ± SD 7.23) (Table 5).

Significance in mean values in UC patients, in both the above mentioned dimensions, was confirmed as shown in the Table 4; the significant scores for CD patients were found only for the “externally-oriented cognitive style” dimension (mean 21.45 ± SD 8.98) and the score for “difficulty in identifying feelings” reaches a mean of 14.73 (± SD 7.65). No significant statistical differences between the two groups were observed (*p* > 0.05).

**Table 4:** Psychopatological and alexithymia scores analysis in sub-group UC/CD and comparative analysis using the Wilcoxon-Mann-Whitney test (*p* < 0.01).

	**UC**		**CD**					
	**Mean**	**± SD**	**Mean**	**± SD**	**U**	**W**	**Z**	***P***
HADS Anxiety	7.15	4.22	7.2	7.15	3206	8156	−0.04	0.97
HADS Depression	4.9	3.5	5.8	5.8	2838.05	7788.5	−1.28	0.2
SCL-90-R somatisation	0.82	0.7	1.04	0.92	857	4685	−7.26	<0.001
SCL-90-R obsessive compulsive	0.91	0.71	1.2	1.17	1068.5	4895.5	−6.466	<0.001
SCL-90-R depression	0.83	0.75	1.39	1.09	544	4372	−8.439	<0.001
SCL-90-R global severity index	0.7	0.55	1.15	0.75	453	4281	−8.778	<0.001
TAS-20 “difficulty identifying feelings”	15.41	6.9	14.73	7.65	114.55	5027.5	−0.95	0.34
TAS-20 “difficulty describing feelings”	9.87	4.18	10	4.33	3006.5	5284.5	−0.03	0.98
TAS-20 “externally oriented cognitive style”	18.69	5.88	21.45	8.98	2489.5	6584.5	−1.868	0.062
TAS-20 total score	44.1	13.66	45.9	15.06	2878	6973	−0.486	0.627

**Table 5:** Psychopatological and alexithymia scores analysis in the IBD patients group.

	**Mean**	**± SD**
HADS Anxiety	7.17	4.34
HADS Depression	5.26	3.82
SCL-90-R somatisation	0.87	0.76
SCL-90-R obsessive compulsive	0.97	0.82
SCL-90-R depression	0.94	0.85
SCL-90-R global severity index	0.78	0.62
TAS-20 “difficulty identifying feelings”	15.12	7.23
TAS-20 “difficulty describing feelings”	9.93	4.23
TAS-20 “externally-oriented cognitive style”	19.89	7.48
TAS-20 total score	44.87	14.26

### Correlations between psychopathology, alexithymia, socio-demographic variables, and CD/UC variables (Table 6)

Considering the socio-demographic variables, high levels of anxiety were more frequently reported in women (HADS anxiety rs 0.18; *p* = 0.02), while significant levels of depression were more common in older patients (HADS depression rs 0.179; *p* = 0.021). A positive correlation was found between IBD-type (CD > UC) and somatisation (rs 0.568; *p* < 0.001) and obsessive-compulsive symptoms (rs 0.506; *p* < 0.001), depression (rs 0.678; *p* < 0.001), anxiety (rs 0.550; *p* < 0.001), and global severity index (rs 0.712; *p* < 0.001).

**Table 6:** Correlation between socio-demographic and clinical variables and scores of rating scales using Spearman's correlation (*p* < 0.05).

		**Rho Spearman**	***P***
Age	HADS Depression	0.179	0.021
Gender (F)	HADS Anxiety	0.18	0.02
Diagnosis (CD)	SCL-90-R somatisation	0.568	<0.001
	SCL-90-R obsessive compulsive	0.506	<0.001
	SCL-90-R depression	0.678	<0.001
	SCL-90-R anxiety	0.550	<0.001
	SCL-90-R global severity index	0.712	<0.001
IBD extension	SCL-90-R somatisation	0.32	<0.001
	SCL-90-R obsessive compulsive	0.402	<0.001
	SCL-90-R depression	0.471	<0.001
	SCL-90-R anxiety	0.389	<0.001
	SCL-90-R global severity index	0.456	<0.001
	TAS-20 total score	0.16	0.05
Delay of diagnosis	HADS Depression	0.294	0.002
	SCL-90-R somatisation	0.306	0.002
	SCL-90-R depression	0.252	0.012
	SCL-90-R anxiety	0.244	0.015
	SCL-90-R global severity index	0.251	0.012
	TAS-20 total score	0.213	0.033
Surgery	HADS Depression	0.192	0.016
	HADS Anxiety	0.276	0.007
	SCL-90-R somatisation	0.168	0.043
	SCL-90-R obsessive compulsive	0.263	0.001
	SCL-90-R depression	0.295	<0.001
	SCL-90-R anxiety	0.248	0.001
	SCL-90-R global severity index	0.267	0.001
Poly-therapy	TAS-20 total score	0.2	0.01
Level of Alexithymia (TAS 20 total score)	HADS Anxiety	0.516	<0.001
	HADS Depression	0.556	<0.001
	SCL-90-R somatisation	0.229	0.005
	SCL-90-R obsessive compulsive	0.362	<0.001
	SCL-90-R depression	0.274	0.001
	SCL-90-R global severity index	0.265	0.001

Furthermore, the extension of IBD positively correlates with somatisation symptoms (rs 0.32; *p* < 0.001), obsessive-compulsive symptoms (rs 0.402; *p* < 0.001), depression (rs 0.471; *p* < 0.001), anxiety (rs 0.389; *p* < 0.001), and global severity symptoms (rs 0.456; *p* < 0.001). Diagnostic delay and previous surgical treatment were positively associated with depressive symptoms in the HADS screening (rs 0.294; *p* = 0.002—rs 0.192; *p* = 0.016) and in the assessment with SCL-90-R (rs 0.252; *p* = 0.012—rs 0.295; *p* < 0.001), somatisation symptoms (rs 0.306; *p* = 0.002—rs 0.168; *p* = 0.043), anxiety (rs 0.244; *p* = 0.015—rs 0.248; *p* = 0.001), and global severity index (rs 0.251; *p* = 0.012—rs 0.267; *p* = 0.001). A history of surgery for IBD was associated with a higher prevalence of obsessive-compulsive symptoms (rs 0.263; *p* = 0.001).

The total values of alexithymia on the TAS-20 scale had a positive correlation with diagnostic delay (rs 0.213; *p* = 0.033), utilization of IBD-specific poly-therapies (rs 0.200; *p* = 0.01), and IBD extension (rs 0.16; *p* = 0.05).

The depressive symptoms evaluated both through the HADS scale (rs 0.556; *p* < 0.001) and the SCL-90-R scale (rs 0.274; *p* = 0.001), together with anxiety (rs 0.516; *p* < 0.001), somatisation symptoms (rs 0.229; *p* = 0.005), obsessive-compulsive symptomatology (rs 0.362; *p* < 0.001), and general severity index (rs 0.265; *p* = 0.001) correlated positively with the global alexithymia level.

## Discussion

In our outpatient sample, although made of clinically remitted IBD patients, a high prevalence of anxious, depressive symptoms, and alexithymia was confirmed, according to the several data in literature (34, 48–50).

The study of the two sub-populations brings out differences in the psychopathologic phenotype. Even while manifesting similar levels of anxiety, patients affected by CD are usually more severe than those with UC, manifesting depressive symptoms with somatisations and symptoms on the OC spectrum (control, rumination). The close relation between anxious-depressive symptoms and IBD is presently explained in the emerging theories of “gut-brain axis” dysfunction (51, 52). The presence of an alteration of the immune system in pro-inflammatory sense involves an alteration of the micro-anatomy and the gut microbiota (53), promoting the development of a neuro-inflammation, with a modification of synaptic plasticity and neuronal functioning (54, 55). Overall, findings seem to confirm data emerging from a recent meta-analysis showing correlation between depressive symptoms and the negative progression of the disease, particularly in CD patients, who are more likely to suffer from depression compared with UC patients (56). The correlation between the greater extension of the disease and psychopathological severity reinforces the already-discussed differences relative to the group of patients suffering from CD. The evidence of correlation between depressive symptoms and surgical interventions (57, 58) was confirmed and was shown to be more frequent in patients of CD, in comparison to those affected by UC, causing worse effects on the quality of life. These symptoms could explain the greater use of antidepressant medications in patients suffering from CD (59). Another significant datum regards the impact of diagnostic delay on psychiatric symptoms: in the sample examined, it seems to overlap previous data (60). The beginning of an adequate therapeutic process might constitute a factor of important psycho-physic stress with a subsequent impact on the symptomatological level. In the same way, the already-evident correlation between anxiety symptoms vs. female gender and depressive symptoms vs. old age had been already highlighted in analogous studies dedicated to the populations affected by IBD (61).

The studies of the prevalence of alexithymia in patients suffering from chronic diseases (62) suggest a close relationship with anxious and depressive clinical features. A significant presence of alexithymia can be identified also in patients from the sample affected by IBD, with no significant difference between the two groups of patients. The global level of alexithymia and all the dimensions of the construct correlate with a greater severity of psychiatric symptomatology, especially in the already-significant areas of depression, anxiety, somatisation, and obsessive-compulsive symptoms.

The association between alexithymia and obsessive-compulsive symptoms is well-known (63, 64). Some studies identified alexithymic construct as an endo-phenotype associated with pure obsessions (in particular “difficulty in describing emotions”) and hoarding and checking compulsions (27). In alexithymic patients, the OC symptoms would configure as maladaptive coping strategies for negative emotions that the patient is not able to comprehend and elaborate on. Furthermore, in a recent publication Filipovic et al. (65) suggest that it would be desirable to deepen the possible comorbidity with C-cluster personality traits in patients affected by IBD (which included the obsessive-compulsive personality disorder), underlining some evidence already pointed out in the case of other chronic diseases.

The presence of somatic symptoms in the general population of alexithymic patients is well known and is basically associated to a worsening of painful syndromes (66, 67) and also to a worse condition in the forms of functional intestinal syndromes (68). The presence of “difficulty in identifying emotions” and “externally-oriented thinking” are associated with a reduced quality of life with a high correlation to anxious and depressive symptoms, somatisations, and reduced social functioning (69–71). From our data, alexithymia is also associated with a broader extension of gastrointestinal involvement and to a longer duration of undiagnosed pathology, which, as already seen, are in turn associated with a greater symptomatological severity. This evidence could be explained by the “stress-alexithymia hypothesis,” according to which elevated levels of alexithymia are associated to an immune system alteration in a pro-inflammatory sense (72). As already discussed, a higher level of acute-phase proteins may predispose patients to neuro-inflammation mediated psychiatric symptoms while, on the other hand, they could worsen the course of the gastrointestinal pathology. The alexithymic patients could be unable to express the suffering related to the onset of the disease, favoring the delay of the diagnosis. Moreover, the alexithymic patients examined are predisposed to the use of poly-therapies: this is likely, as these are patients who reached a clinical remission after a long period of treatments in which mono-therapies had failed, and can thus be defined as a population of patients who is poorly responsive to medical treatments.

Because alexithymia is a mental state denoting the inability to identify emotions at a cognitive level, one hypotesis is that IBD patients expressing high levels of alexithymia could misattribute autonomic symptoms of anxiety (for example diarrhea and abdominal pain) to that of IBD.

As described above and in a previous study (73), there are no differences in alexithymia levels between CD and UC patients, suggesting that this cognitive construct could be a common trait that is globally oriented to worse mental health and poor clinical outcome, leading patients to communicate their distress through somatic symptoms rather than verbal contents.

## Ethics and dissemination

The actual scientific evidence, within which the resulting data are convincingly placed, increasingly underline the necessity for a screening (74, 75) for psychiatric symptoms in patients suffering from IBD, especially for CD patients. The early identification of subjects with significant symptoms must be taken into account to activate adequate treatment processes (76, 77) in the perspective that those who do not receive adequate treatment might be at higher risk of relapses and a more aggressive form of the disease (78). In order to implement the psychological and psychiatric support and treatment of IBD patients, the “Ge.Co—Gastroenterologist collaboration project” has been active in our Hospital since 2016. This project includes different professionals interacting to create an integrated exchange of competences. Patients can therefore take advantage of gastroenterological, surgical together with psychiatric check-ups, as well as psychological counseling with cognitive-behavioral orientation, for the most part based on the mindfulness approach (79). In our experience, in less than 2 years the GE.Co Project has involved about 50 patients which have been receiving both pharmacological and psychological treatment. Our further goal is to extend participation in the project to the greatest number of IBD patients, in order to improve the diagnostic process in order to recognize specific anxiety or mood disorders according to DSM5 criteria and to begin prompt treatment.

A limitation of this study is the lack of data regarding possible risk factors for depression and anxiety, such as life events and patients' socioeconomic level, and the effect of these factors in relation to compliance with the treatments. Furthermore, this study is a correlational study performed in a naturalistic setting, which has screened for psychopathology symptoms. Further longitudinal studies are necessary to highlight the prevalence of psychiatric disorders in a representative sample of patients including those with active disease.

## Author contributions

CV and GM: study conception and design; MMB and MV: study execution; MMB: analysis and interpretation of data; MMB, MFB, CV, and GM: contribution in the discussion and analysis; MMB, CV, and RZ: drafting of paper.

### Conflict of interest statement

The authors declare that the research was conducted in the absence of any commercial or financial relationships that could be construed as a potential conflict of interest.
